# Angiotensin-II-derived reactive oxygen species on baroreflex sensitivity during hypertension: new perspectives

**DOI:** 10.3389/fphys.2013.00105

**Published:** 2013-05-13

**Authors:** Thyago M. de Queiroz, Matheus M. O. Monteiro, Valdir A. Braga

**Affiliations:** Department of Biotechnology, Biotechnology Center, Federal University of ParaibaJoão Pessoa, Brazil

**Keywords:** hypertension, antioxidants, angiotensin-II, NADPH oxidase, baroreflex

## Abstract

Hypertension is a multifactorial disorder, which has been associated with the reduction in baroreflex sensitivity (BRS) and autonomic dysfunction. Several studies have revealed that increased reactive oxygen species (ROS) generated by nicotinamide adenine dinucleotide phosphate [NAD(P)H] oxidase, following activation of type 1 receptor (AT_1_R) by Angiotensin-(Ang) II, the main peptide of the Renin–Angiotensin–Aldosterone System (RAAS), is the central mechanism involved in Ang-II-derived hypertension. In the present review, we will discuss the role of Ang II and oxidative stress in hypertension, the relationship between the BRS and the genesis of hypertension and how the oxidative stress triggers baroreflex dysfunction in several models of hypertension. Finally, we will describe some novel therapeutic drugs for improving the BRS during hypertension.

## Angiotensin-II is involved in hypertension

Angiotensin-(Ang) II is the key peptide of the Renin–Angiotensin–Aldosterone System (RAAS). This system consists mainly of an enzymatic cascade catalyzed by renin and angiotensin converting enzyme (ACE), generating Ang II (Peach, 1977; Griendling et al., 1993). The effect of Ang II is mediated by Ang II receptors. Two isoforms of the Ang II receptor have been identified: type 1 receptor (AT_1_R) and type 2 receptor (AT_2_R).

Ang II and its receptors have multiple systemic and local actions in different tissues, including blood vessels, kidneys, adrenal glands, heart and central nervous system (CNS) (Sadjadi et al., 2002; Campos, 2009; Campos et al., 2011). For instance, in the vasculature, activation of AT_1_R induces potent vasoconstriction (Ito et al., 1995; Oliverio et al., 1997). In the adrenal glands, their activation stimulates the release of aldosterone that in turn promotes sodium reabsorption in the mineralocorticoid-responsive segments of the distal nephron (Masilamani et al., 1999). In the kidneys, activation of AT_1_R is associated with renal vasoconstriction and antinatriuresis (Navar et al., 1987). Furthermore, AT_1_R is involved in the progression of cardiovascular diseases including hypertension, atherosclerosis, cardiac hypertrophy, and heart failure (Stegbauer and Coffman, 2011; Ichiki et al., 2012).

Additionally, expression of the AT_2_R increases under pathological situations (Li et al., 2005; Padia and Carey, 2013). Therefore, activation of AT_2_R triggers nitric oxide (NO) release (Herrera and Garvin, 2010) and inhibits NF-κB and JAK/STAT signaling pathways. Thus, AT_2_R effects would potentially neutralize those of AT_1_R leading to cardiovascular protection. Moreover, AT_2_R activation directly antagonizes AT_1_R mediated actions (Horiuchi et al., 1999; Stegbauer and Coffman, 2011).

Regarding Ang II effects on the CNS, a high density of Ang II type 1 receptors was found in specific regions of the forebrain and in the rostral ventrolateral medulla (RVLM) (Allen et al., 1998). Of note, microinjection of Ang II into the RVLM produces an AT_1_R-mediated increase in autonomous nervous system, resulting in increases in blood pressure (Dampney et al., 2007). Furthermore, the overexpression of AT_1_R in the RVLM increases blood pressure (Allen et al., 2006), and the blockade of AT_1_R in the RVLM has been associated to reduction blood pressure in several forms of experimental hypertension (Ito et al., 2002; Braga, 2010). Recently, using combined *in vivo* and molecular biology approaches, we have documented that Ang II-induced hypertension is mediated by an increase in sympathetic nerve activity, which seems to involve up-regulation of AT1 receptors in the RVLM and down-regulation of AT1 receptors in the subfornical organ (SFO) (Braga, 2011; Nunes and Braga, 2011).

An additional component of the RAAS family, angiotensin converting enzyme 2 (ACE2) cleaves Ang I and Ang II into Ang-(1–9) and Ang-(1–7), respectively (Chang et al., 2011). Ang-(1–7) has opposite properties to that of Ang II. By acting through the Mas receptor, Ang-(1–7) promotes vasodilation, antiproliferation, and antihypertrophy (Santos et al., 2003; Ferrario et al., 2005). In the brain, Ang-(1–7) was reported to produce depressor responses when administered in the nucleus of the tractus solitarius (NTS) and dorsal motor nucleus of the vagus nerve (Campagnole-Santos et al., 1989). There is compelling evidence that ACE2 may play a pivotal role in counterbalancing the undesirable actions of the ACE/Ang II/AT_1_R axis and may be beneficial for the cardiovascular system (Xu et al., 2011).

## Oxidative stress in hypertension

The role of oxidative stress in the generation and/or maintenance of arterial hypertension has recently been reported in various animal models of hypertension, including the renovascular two-kidney–one-clip model (2K1C) (Oliveira–Sales et al., 2008; Botelho-Ono et al., 2011; Burmeister et al., 2011), the one-kidney–one-clip hypertension model (1K1C) (Prewitt et al., 2001), the Ang II-induced hypertension model (Zimmerman et al., 2002, 2004a; Laplante et al., 2003; Braga, 2011; Nunes and Braga, 2011), the Dahl salt-sensitive (DOCA-salt) hypertension model (Wu and De Champlain, 1999; Braga, 2010) the spontaneously hypertensive rat model (SHR) (Nishikawa et al., 2003; de Champlain et al., 2004; Guimarães et al., 2012; Monteiro et al., 2012) and the stroke-prone SHR model (SHRSP) (Chen et al., 2001; Kishi et al., 2004) as well as in humans (Duffy et al., 1999; Higashi et al., 2002; Campos et al., 2011).

For example, increased reactive oxygen species (ROS), including superoxide anion formation precedes the development of hypertension in SHR and in Ang II-infused mice (Kitiyakara and Wilcox, 1998; Houston, 2005; Braga et al., 2008; Botelho-Ono et al., 2011).

More than a decade ago, Griendling et al. (1994) first discovered that Ang II activates the vascular smooth muscle nicotinamide adenine dinucleotide phosphate [NAD(P)H] oxidase, an important cellular source of ROS (Griendling et al., 1994). Subsequently, it was shown that hypertension caused by Ang II infusion, but not norepinephrine infusion, increased vascular superoxide production *in vivo* (Rajagopalan et al., 1996) and that adenovirus-mediated superoxide dismutase (SOD) overexpression was effective in preventing this form of hypertension (Laursen et al., 1997; Zimmerman et al., 2004b; Davisson and Zimmerman, 2010; Lob et al., 2010).

The NAD(P)H oxidase is a multi-subunit enzyme and is one of the enzymatic sources of superoxide production. The NAD(P)H oxidase has five subunits: p47phox (“phox” stands for phagocyte oxidase), p67phox, p40phox, p22 phox, and the catalytic subunit gp91phox (also termed “Nox2”) (Chabrashvili et al., 2002; Babior, 2004). In unstimulated cells, p47phox, p67phox, and p40phox are located in the cytosol, whereas p22phox and gp91phox are in the membrane (Touyz et al., 2003). Upon stimulation, p47phox becomes phosphorylated and the cytosolic subunits form a complex that translocates to the membrane and activates the NAD(P)H oxidase complex (Touyz et al., 2003; Campos et al., 2011).

To fully understand how NADPH oxidase is involved in the context of neurogenic hypertension, and to be able to target it precisely, either experimentally or therapeutically, information about the expression patterns of the Nox homologues is required. To this end, Infanger et al. (2006) compared the expression levels of Nox1, Nox2, and Nox4 in different regions of mouse brain using real-time PCR. Their data showed that Nox2 as well as Nox4 are the predominant homologues expressed in fore-, mid-, and hind-brain of mice, while Nox1 is detectable but at very low levels. One limitation is that, in a variety of cell types, Nox transcript levels at baseline do not necessarily predict stimulus-induced activation of the enzymes, and opposing functions of various enzymes have been detected under different physiological conditions. Taken together, it is possible to suggest that an increase in NADPH oxidase-derived ROS in circumventricular organs CVOs, hypothalamic nuclei, and brainstem sites play a central role in the neurocardiovascular dysfunction observed in hypertension (Braga et al., 2011a,b).

Accumulating evidence now points to oxidative stress as a key mechanism in Ang II-dependent neurogenic hypertension (Kitiyakara and Wilcox, 1998; Houston, 2005; Burmeister et al., 2011). Inhibition of the NAD(P)H oxidase, with a decreased in oxidative stress, in CVOs of brain such as the SFO attenuated the cardiovascular and dipsogenic effects to intracerebroventricular (ICV) administration of Ang II (Zimmerman et al., 2004a; Peterson et al., 2009). Similar effect was observed in paraventricular nucleus of the hypothalamus (PVN) (Burmeister et al., 2011) and RVLM (Braga et al., 2008; Braga, 2010). More recently, Chrissobolis et al. (2012) have documented that Nox2 appears to be the more prominent mediator of the harmful effects of Ang II in the cerebral circulation during hypertension. In addition, injections of adenoviral vectors expressing small interfering (si)RNA targeting NOX2 (AdsiRNA-NOX2) or NOX4 (AdsiRNA-NOX4) mRNAs, used to knock down NOX2 and NOX4 proteins, in the PVN showed that either AdsiRNA-NOX2 or AdsiRNA-NOX4 significantly attenuated the development of Aldo/NaCl-induced hypertension (Xue et al., 2012). In an additional study by the same group, Aldo/salt-induced hypertension was also significantly attenuated in NOX2 (genomic) knockout mice compared with wild-type controls. When animals from both functional studies underwent ganglionic blockade, there was a reduced fall in blood pressure in the NOX2 and NOX4 knockdown/knockout mice, indicating that both NOX2 and NOX4 in the PVN contribute to hypertension (Xue et al., 2012).

In addition to ROS, reactive nitrogen species play an important role in the pathogenesis of hypertension. NO and peroxinitrite, their main players, have been reviewed elsewhere (Pacher et al., 2007). Briefly, the formation of reactive nitrogen species is a consequence of NO synthesis. Accumulating evidence suggests that alterations in NO synthesis and NO-sGC-cGMP signaling or a reduction in the bioavailability of endothelium-derived NO by increased oxidative stress are key contributors to the pathogenesis of hypertension (Wolin, 2005; Paravicini and Touyz, 2006). Increased levels of superoxide have been shown to decrease the bioavailability of NO, thereby contributing to the maintenance of elevated peripheral resistance (Cai and Harrison, 2000; Ungvari et al., 2004). NO is efficiently removed by reacting with oxyhemoglobin to form nitrate, which prevents even the highest rates of NO synthesis from directly reacting with oxygen to form significant amounts of nitrogen dioxide. However, the simultaneous activation of superoxide synthesis along with NO will transform the biological actions of NO by forming peroxynitrite (Pacher et al., 2007). Several enzyme complexes, including NADPH oxidases and xanthine oxidase, can be activated in many cellular systems to actively produce significant amounts of superoxide. When superoxide and NO are produced simultaneously in close proximity, modestly increasing superoxide and NO each at a 10-fold will increase peroxynitrite formation by 100-fold. Without superoxide, the formation of nitrogen dioxide by the reaction of NO with oxygen is miniscule by comparison (Pacher et al., 2007). NO and superoxide do not even have to be produced within the same cell to form peroxynitrite, because NO can so readily move through membranes and between cells. Although peroxynitrite is a strong oxidant, it reacts at a relatively slow rate with most biological molecules. Compelling evidence has emerged supporting the importance of endogenous peroxynitrite formation and protein nitration in the pathogenesis of arterial hypertension [reviewed in details by Turko and Murad (2002)].

## Baroreflex and antioxidants

The arterial baroreflex, regulated by the CNS acts to oppose the increase in blood pressure by inhibiting sympathetic activity, causing vasodilation and reducing heart rate (HR) in the short term. Previous reports have suggested that baroreflex sensitivity (BRS) is reduced during hypertension and the mechanisms underlying its reduction involves ROS (Braga, 2010; Botelho-Ono et al., 2011; Guimarães et al., 2012; Queiroz et al., 2012).

In recent decades, several research groups have worked extensively to improve the treatment of hypertension and its complication, including reduction in BRS, focusing on the discovery of new therapy strategies and drugs (Lefkowits and Willerson, 2001; Queiroz et al., 2011). Among these, we can highlight the use of antioxidant therapy, such as ROS scavengers and vitamins, SOD mimetics or NAD(P)H oxidase inhibitors that has experimentally shown to attenuate or prevent the development of hypertension (Chen et al., 2001; Landmesser et al., 2003; Costa et al., 2009; Queiroz et al., 2012).

It has been reported that microinjection of tempol into the RVLM decreased mean arterial pressure (MAP) and HR in SHRSP but not in WKY (Kishi and Hirooka, 2013). Other study demonstrated that acute intravenous infusion of ascorbic acid (Vitamin C) restores the reduced BRS in renovascular hypertension and that the inhibition of the NAD(P)H oxidase also restores BRS in hypertensive animals (Botelho-Ono et al., 2011). Moreover, it has been recognized that chronic administration of vitamin C for seven days improves BRS in renovascular hypertensive rats (Nishi et al., 2010).

Studies performing ICV injections of adenovirus encoding SOD (AdCuZnSOD) in SFO or RVLM demonstrated that the pressor effects caused by Ang II infusion were attenuated. Girouard et al. (2004) found that NAC or melatonin treatment in drinking water increased baroreflex control in response to pressor and depressor stimulations in SHR. In addition, the treatment enhanced increase basal plasma norepinephrine levels in hypertensive. Guimarães et al. (2012) showed that both acute intravenous administration of tiron, a SOD mimetic, and apocynin, a NAD(P)H oxidase inhibitor, induce reductions in oxidative stress triggering improvement in BRS in SHR.

Studies aiming to characterize the signal transduction mechanism of PI3-kinase involvement in Ang II-induced stimulation of central neuronal activity in cultured neurons and Ang II-induced inhibition of baroreflex in SHR vs. WKY rats showed that application of Ang II to neurons produced a 42% greater increase in neuronal firing in cells from the SHR compared to WKY. Interestingly, the Ang II-mediated increase in firing rate was abolished entirely by the PKC inhibitor GF109230 in the WKY while it was necessary to block both PKC and PI3K activity to produce the same effect in the SHR (Sun et al., 2009). This was associated with an increased ability of Ang II to stimulate NADPH-oxidase-ROS mediated signaling involving phosphorylation of the p47phox subunit of the NADPH oxidase and was dependent on the activation of PI3 Kinase in the SHR. In addition, inhibition of PI3 Kinase resulted in the reduction of levels of p47phox phosphorylation, NADPH oxidase activity, ROS levels and ultimately neuronal activity in cells from the SHR but not the WKY rat. In addition, in working heart-brainstem preparations, inhibition of PKC activity in the NTS *in situ* abolished the Ang II-mediated depression of cardiac and sympathetic baroreceptor reflex gain in the WKY. In contrast, PKC inhibition in the NTS of SHR only partially reduced the effect of Ang II on the baroreceptor reflex gain (Sun et al., 2009).

Furthermore, aiming to determine whether or not chronic reduction of ROS in the RVLM improves impaired BRS in hypertensive rats, Ogawa et al. (2012) transfected adenovirus vectors encoding either manganese superoxide dismutase (AdMnSOD) or β-galactosidase (AdLacZ) into the RVLM of SHRSP and measured BRS using the spontaneous sequence method. They reported that BRS was significantly lower in SHRSPs than in WKY rats. In addition, in AdMnSOD-transfected SHRSP, blood pressure, HR, and sympathetic nervous system activation were significantly decreased from day 5 after the gene transfer. In contrast, BRS in the AdMnSOD-transfected SHRSP was significantly increased from day 4 after the gene transfer with the reduction of ROS in the RVLM. Furthermore, in the AdMnSOD-transfected SHRSP, intravenous infusion of atropine dramatically decreased BRS. In contrast, in the AdLacZ-transfected SHRSP, atropine did not decrease BRS. Their results suggest that chronic reduction of ROS in the RVLM improves the impaired BRS in SHRSP through inhibition of the sympathetic component (Ogawa et al., 2012).

## Perspectives on natural products

**Figure 1:**
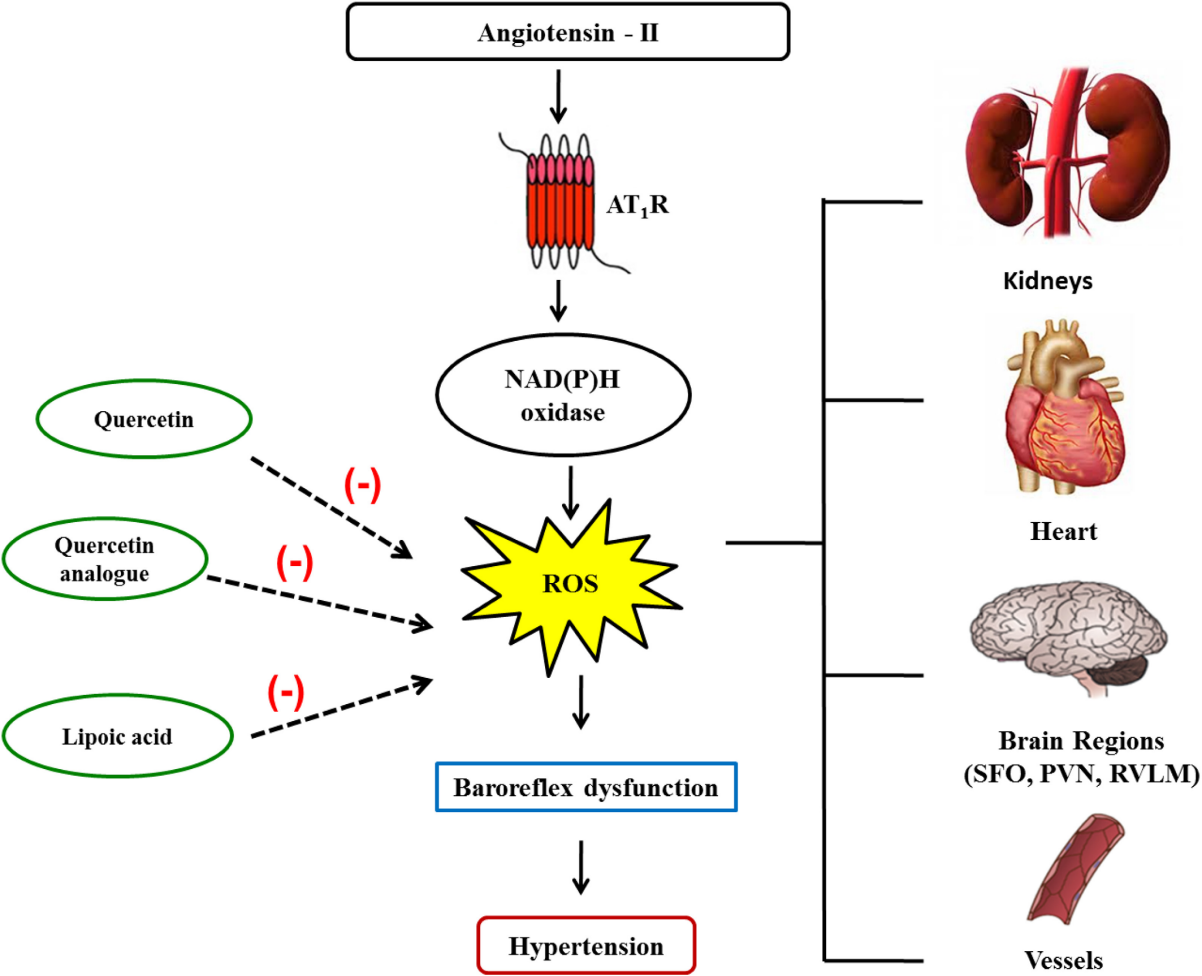
**Angiotensin II and its mechanisms to reduce baroreflex sensitivity.** Angiotensin II binds to its receptors activating NADPH oxidase, which in turn increases reactive oxygen species (ROS) generation in several tissues. Within the brain, ROS leads to a reduction in the baroreflex sensitivity, which contributes to hypertension. Natural products such as quercetin, quercetin analogs, and alpha-lipoic acid, due to their antioxidant capability, improve baroreflex function, and ameliorate hypertension.

Studies from our laboratory revealed that natural products were capable of improving BRS through ROS scavenging mechanisms. The flavonoid quercetin improves both sympathetic and parasympathetic components of baroreflex and reduces MAP in SHR due to reduction of systemic oxidative stress. The mechanism is unknown, but it was suggested that this antioxidant can interfere with three or more different free radical–producing systems (Monteiro et al., 2012). Other study from our group showed that antioxidant therapy by chronic treatment with α-lipoic acid, an endogenous antioxidant, reduces hypertension and improves BRS in rats with renovascular hypertension (Queiroz et al., 2012). Preliminary studies with a quercetin analog, rutin, demonstrated that this compound improved cardiovascular parameters altered during hypertension such as BRS and vascular reactivity, probably by a reduction in oxidative stress (Figure 1).

In conclusion, natural products with antioxidant properties have emerged as new potential therapeutic tools for improving BRS during hypertension. Although promising, there is still a long way until their use in the clinics becomes reality.

### Conflict of interest statement

The authors declare that the research was conducted in the absence of any commercial or financial relationships that could be construed as a potential conflict of interest.

## References

[B1] Allen A. M. Dosanjh J. K. Erac M. Dassanayake S. Hannan R. D. Thomas W. G. (2006). Expression of constitutively active angiotensin receptors in the rostral ventrolateral medulla increases blood pressure. Hypertension 47, 1054–1061 10.1161/01.HYP.0000218576.36574.54 16618838

[B2] Allen A. M. Moeller I. Jenkins T. A. Zhuo J. Aldred G. P. Chai S. Y. (1998). Angiotensin receptors in the nervous system. Brain Res. Bull. 47, 17–28 10.1016/S0361-9230(98)00039-2 9766385

[B3] Babior B. M. (2004). NADPH oxidase. Curr. Opin. Immunol. 16, 42–47 10.1016/j.coi.2003.12.001 14734109

[B4] Botelho-Ono M. S. Pina H. V. Sousa K. H. F. Nunes F. C. Medeiros I. A. Braga V. A. (2011). Acute superoxide scavenging restores depressed baroreflex sensitivity in renovascular hypertensive rats. Auton. Neurosci. 159, 38–44 10.1016/j.autneu.2010.07.025 20719579

[B5] Braga V. A. (2010). Dietary salt enhances angiotensin-II-induced superoxide formation in the rostral ventrolateral medulla. Auton. Neurosci. 155, 14–18 10.1016/j.autneu.2009.12.007 20053589

[B6] Braga V. A. (2011). Differential brain angiotensin-II type I receptor expression in hypertensive rats. J. Vet. Sci. 12, 291–293 21897104 10.4142/jvs.2011.12.3.291 PMC3165160

[B7] Braga V. A. Burmeister M. A. Zhou Y. Sharma R. V. Davisson R. L. (2008). Selective ablation of AT1a receptors in rostral ventrolateral medulla (RVLM) prevents chronic angiotensin-II-dependent hypertension in part by reducing oxidant stress in this region. Hypertension 52, 36

[B8] Braga V. A. Colombari E. Jovita M. G. (2011a). Angiotensin II-derived reactive oxygen species underpinning the pro-cessing of the cardiovascular reflexes in the medulla oblongata. Neurosci. Bull. 27, 269–274 10.1007/s12264-011-1529-z 21788998 PMC5560308

[B9] Braga V. A. Medeiros I. A. Ribeiro T. P. França-Silva M. S. Botelho-Ono M. S. Guimarães D. D. (2011b). Angiotensin-II-derived reactive oxygen species along the SFO-PVN-RVLM pathway: implications in neurogenic hypertension. Braz. J. Med. Biol. Res. 44, 814–965 10.1590/S0100-879X2011007500088 21755262

[B10] Burmeister M. A. Young C. N. Braga V. A. Butler S. D. Sharma R. V. Davisson R. L. (2011). *In vivo* bioluminescence imaging reveal redox-regulated activator protein-1 activation in paraventricular nucleus of mice with renovascular hypertension. Hypertension 57, 289–297 10.1161/HYPERTENSIONAHA.110.160564 21173341 PMC3026319

[B11] Cai H. Harrison D. G. (2000). Endothelial dysfunction in cardiovascular diseases: the role of oxidant stress. Circ. Res. 87, 840–844 10.1161/01.RES.87.10.840 11073878

[B12] Campagnole-Santos M. J. Diz D. I. Santos R. A. Khosla M. C. Brosnihan K. B. Ferrario C. M. (1989). Cardiovascular effects of angiotensin-(1–7) injected into the dorsal medulla of rats. Am. J. Physiol. Heart. Circ. Physiol. 257, 324–329 2750946 10.1152/ajpheart.1989.257.1.H324

[B13] Campos R. R. (2009). Oxidative stress in the brain and arterial hypertension. Hypertens. Res. 32, 1047–1048 10.1038/hr.2009.180 19893566

[B14] Campos R. R. Oliveira-Sales E. B. Nish E. M. Boim M. A. Dolnikoff M. S. Bergamaschi C. S. (2011). The role of oxidative stress in renovascular hypertension special series: stress and hypertension. Clin. Exp. Pharmacol. Physiol. 38, 144–152 10.1111/j.1440-1681.2010.05437.x 20678153

[B15] Chabrashvili T. Tojo A. Onozato M. L. Kitiyakara C. Quinn M. T. Fujita T. (2002). Expression and cellular localization of classic NADPH oxidase subunits in the spontaneously hypertensive rat kidney. Hypertension 39, 269–274 10.1161/hy0202.103264 11847196

[B15a] Chang S. Y. Chen Y. W. Chenier I. Tran Sle M. Zhang S. L. (2011). Angiotensin II type II receptor deficiency accelerates the development of nephropathy in type I diabetes via oxidative stress and ACE2. Exp. Diabetes Res. 2011:521076 10.1155/2011/521076 22110472 PMC3205615

[B16] Chen X. Touyz R. M. Park J. B. Schiffrin E. L. (2001). Antioxidant effects of vitamins C and E are associated with altered activation of vascular NADPH oxidase and superoxide dismutase in stroke-prone SHR. Hypertension 38, 606–611 10.1161/hy09t1.094005 11566940

[B17] Costa C. A. Amaral T. A. Carvalho L. C. Ognibene D. T. da Silva A. F. Moss M. B. (2009). Antioxidant treatment with tempol and apocynin prevents endothelial dysfunction and development of renovascular hypertension. Am. J. Hypertens. 22, 1242–1249 10.1038/ajh.2009.186 19779472

[B18] Chrissobolis S. Banfi B. Sobey C. G. Faraci F. M. (2012). Role of Nox isoforms in angiotensin II-induced oxidative stress and endothelial dysfunction in brain. J. Appl. Physiol. 113, 184–191 10.1152/japplphysiol.00455.2012 22628375 PMC3774474

[B19] Dampney R. A. Tan P. S. Sheriff M. J. Fontes M. A. Horiuchi J. (2007). Cardiovascular effects of angiotensin II in the rostral ventrolateral medulla: the push–pull hypothesis. Curr. Hypertens. Rep. 9, 222–227 17519129 10.1007/s11906-007-0040-4

[B20] Davisson R. L. Zimmerman M. C. (2010). Angiotensin, II, oxidant signaling, and hypertension: down to a T? Hypertension 55, 228–230 10.1161/HYPERTENSIONAHA.109.144477 20008674 PMC2811261

[B21] de Champlain J. Wu R. Girouard H. Karas M. Midaoui A. E. Laplante M. A. (2004). Oxidative stress in hypertension. Clin. Exp. Hypertens. 8, 593–601 15702613 10.1081/ceh-200031904

[B22] Duffy S. J. Gokce N. Holbrook M. Huang M. Frei B. keaney J. F. (1999). Treatment of hypertension with ascorbic acid. Lancet 354, 2048–2049 10636373 10.1016/s0140-6736(99)04410-4

[B23] Ferrario C. M. Trask A. J. Jessup J. A. (2005). Advances in the biochemical and functional roles of angiotensin converting enzyme 2 and angiotensin- (1–7) in the regulation of cardiovascular function. Am. J. Physiol. Heart. Circ. Physiol. 289, 2281–2290 10.1152/ajpheart.00618.2005 16055515 PMC7203566

[B24] Girouard H. Denault C. Chulak C. Champlain J. (2004). Treatment by N-acetylcysteine and melatonin increases cardiac baroreflex and improves antioxidant reserve. Am. J. Hypertens. 17, 947–954 10.1016/j.amjhyper.2004.06.009 15485759

[B25] Griendling K. K. Minieri C. A. Ollerenshaw J. D. Alexander R. W. (1994). Angiotensin II stimulates NADH and NADPH oxidase activity in cultured vascular smooth muscle cells. Circ. Res. 74, 1141–1148 10.1161/01.RES.74.6.1141 8187280

[B26] Griendling K. K. Murphy T. J. Alexander R. W. (1993). Molecular biology of the renin-angiotensin system. Circulation 87, 1816–1828 10.1161/01.CIR.87.6.1816 8389259

[B27] Guimarães D. D. Carvalho C. C. Braga V. A. (2012). Scavenging of NADPH oxidase-derived superoxide anions improves depressed baroreflex sensitivity in spontaneously hypertensive rats. Clin. Exp. Pharmacol. Physiol. 39, 373–378 10.1111/j.1440-1681.2012.05679.x 22283703

[B28] Herrera M. Garvin J. L. (2010). Angiotensin II stimulates thick ascending limb NO production via AT(2) receptors and Akt1-dependent nitric-oxide synthase 3 (NOS3) activation. J. Biol. Chem. 285, 14932–14940 10.1074/jbc.M110.109041 20299462 PMC2865342

[B29] Higashi Y. Sasaki S. Nakagawa K. Matsuura H. Oshima T. Chayama K. (2002). Endothelial function and oxidative stress in renovascular hypertension. N. Engl. J. Med. 346, 1954–1962 10.1056/NEJMoa013591 12075056

[B30] Horiuchi M. Hayashida W. Akishita M. Tamura K. Daviet L. Lehtonen J. Y. A. (1999). Stimulation of different subtypes of angiotensin II receptors, AT_1_ and AT_2_ receptors, regulates STAT activation by negative crosstalk. Circ. Res. 84, 876–882 10.1161/01.RES.84.8.876 10222333

[B31] Houston M. C. (2005). Nutraceuticals, vitamins, antioxidants, and minerals in the prevention and treatment of hypertension. Prog. Cardiovasc. Dis. 47, 396–449 10.1016/j.pcad.2005.01.004 16115519

[B32] Ichikawa I. Brenner B. M. (1980). Importance of efferent arteriolar vascular tone in regulation of proximal tubule fluid reabsorption and glomerulotubular balance in the rat. J. Clin. Invest. 65, 1192–1201 10.1172/JCI109774 7364945 PMC371453

[B33] Ichiki T. Miyazaki R. Kamiharaguchi A. Hashimoto T. Matsuura H. Kitamoto S. (2012). Resveratrol attenuates angiotensin II-induced senescence of vascular smooth muscle cells. Regul. Pept. 177, 35–39 10.1016/j.regpep.2012.04.005 22561451

[B34] Infanger D. W. Sharma R. V. Davisson R. L. (2006). NADPH oxidases of the brain: distribution, regulation, and function. Antioxid. Redox Signal. 8, 1583–1596 10.1089/ars.2006.8.1583 16987013

[B35] Ito M. Oliverio M. I. Mannon P. J. Best C. F. Maeda N. Smithies O. (1995). Regulation of blood pressure by the type 1A angiotensin II receptor gene. Proc. Natl. Acad. Sci. U.S.A. 92, 3521–3525 10.1073/pnas.92.8.3521 7724593 PMC42199

[B36] Ito S. Komatsu K. Tsukamoto K. Kanmatsuse K. Sved A. F. (2002). Ventrolateral medulla AT_1_ receptors support blood pressure in hypertensive rats. Hypertension 40, 552–559 10.1161/01.HYP.0000033812.99089.92 12364362

[B37] Kishi T. Hirooka Y. (2013). Oxidative stress in the brain causes hypertension via sympathoexcitation. Front. Physiol. 3:335 10.3389/fphys.2012.00335 22934082 PMC3429101

[B38] Kishi T. Hirooka Y. Kimura Y. Ito K. Shimokawa H. Takeshita A. (2004). Increased reactive oxygen species in rostral ventrolateral medulla contribute to neural mechanisms of hypertension in stroke-prone spontaneously hypertensive rats. Circulation 109, 2357–2362 10.1161/01.CIR.0000128695.49900.12 15117836

[B39] Kitiyakara C. Wilcox C. S. (1998). Antioxidants for hypertension. Curr. Opin. Nephrol. Hypertens. 7, 531–538 9818200 10.1097/00041552-199809000-00008

[B40] Landmesser U. Dikalov S. Price S. R. Mc-Cann L. Fukai T. Holland S. M. (2003). Oxidation of tetrahydrobiopterin leads to uncoupling of endothelial cell nitric oxide synthase in hypertension. J. Clin. Investig. 111, 1201–1209 10.1172/JCI14172 12697739 PMC152929

[B41] Laplante M. A. Wu R. El Midaoui A. De Champlain J. (2003). NAD(P)H oxidase activation by angiotensin II is dependent on p 42 / 44 ERK-MAPK pathway activation in rats vascular smooth muscle cells. J. Hypertens. 21, 927–936 10.1097/01.hjh.0000059027.82022.6d 12714867

[B42] Laursen J. B. Rajagopalan S. Galis Z. Tarpey M. Freeman B. A. Harrison D. G. (1997). Role of superoxide in angiotensin II–induced but not catecholamine induced hypertension. Circulation 95, 588–593 10.1161/01.CIR.95.3.588 9024144

[B43] Lefkowits R. J. Willerson J. (2001). Prospects for cardiovascular research. JAMA 285, 581–587 10.1001/jama.285.5.581 11176863

[B44] Li J. Culman J. Hortnagl H. Zhao Y. Gerova N. Timm M. (2005). Angiotensin AT_2_ receptor protects against cerebral ischemia induced neuronal injury. FASEB J. 19, 617–619 10.1096/fj.04-2960fje 15665034

[B45] Lob H. E. Marvar P. J. Guzik T. J. Sharma S. McCann L. A. Weyand C. (2010). Induction of hypertension and peripheral inflammation by reduction of extracellular superoxide dismutase in the central nervous system. Hypertension 55, 277–283 10.1161/HYPERTENSIONAHA.109.142646 20008675 PMC2813894

[B46] Masilamani S. Kim G. H. Mitchell C. Wade J. B. Knepper M. A. (1999). Aldosterone-mediated regulation of ENaC α, β, and γ subunit proteins in rat kidney. J. Clin. Invest. 104, 19–23 10.1172/JCI7840 10510339 PMC408561

[B47] Monteiro M. M. O. França-Silva M. S. Alves N. F. B. Porpino S. K. P. Braga V. A. (2012). Quercetin improves baroreflex sensitivity in spontaneously hypertensive rats. Molecules 17, 12997–13008 10.3390/molecules171112997 23117438 PMC6269113

[B48] Navar L. G. Carmines P. K. Huang W. C. Mitchell K. D. (1987). The tubular effects of angiotensin II. Kidney Int. Suppl. 20, 81–88 3298806

[B49] Nishi E. E. Oliveira-Sales E. B. Bergamaschi C. T. Oliveira T. G. Boim M. A. Campos R. R. (2010). Chronic antioxidant treatment improves arterial renovascular hypertension and oxidative stress markers in th e kidney in Wistar rats. Am. J. Hypertens. 23, 473–480 10.1038/ajh.2010.11 20186128

[B50] Nishikawa Y. Tatsumi K. Matsura T. Yamamoto A. Nadamoto T. Urabe K. (2003). Effects of vitamin C on high blood pressure induced by salt in spontaneously hypertensive rats. J. Nutr. Sci. Vitaminol. 49, 301–309 14703303 10.3177/jnsv.49.301

[B51] Nunes F. C. Braga V. A. (2011). Chronic angiotensin II infusion modulates angiotensin II type I receptor expression in the subfornical organ and the rostral ventrolateral medulla in hypertensive rats. J. Renin Angiotensin Aldosterone Syst. 12, 440–445 10.1177/1470320310394891 21393361

[B52] Ogawa K. Hirooka Y. Shinohara K. Kishi T. Sunagawa K. (2012). Inhibition of oxidative stress in rostral ventrolateral medulla improves impaired baroreflex sensitivity in stroke-prone spontaneously hypertensive rats. Int. Heart. J. 53, 193–198 10.1536/ihj.53.193 22790689

[B53] Oliveira–Sales E. B. Dugaich A. P. Abreu N. P. (2008). Oxidative stress supports blood pressure and sympathetic activity in renovascular hypertension. Am. J. Hypertens. 21, 98–104 10.1038/ajh.2007.12 18091751

[B54] Oliverio M. I. Best C. F. Kim H. S. Arendshorst W. J. Smithies O. Coffman T. M. (1997). Angiotensin II responses in AT_1_A receptor-deficient mice: a role for AT_1_B receptors in blood pressure regulation. Am. J. Physiol. 272, 515–520 9140053 10.1152/ajprenal.1997.272.4.F515

[B55] Pacher P. Beckman J. S. Liaudet L. (2007). Nitric oxide and peroxynitrite in health and disease. Physiol. Rev. 87, 315–424 10.1152/physrev.00029.2006 17237348 PMC2248324

[B56] Padia S. H. Carey R. M. (2013). AT2 receptors: beneficial counter-regulatory role in cardiovascular and renal function. Pflugers Arch. 465, 99–110 10.1007/s00424-012-1146-3 22949090 PMC3548020

[B57] Paravicini T. M. Touyz R. M. (2006). Redox signaling in hypertension. Cardiovasc. Res. 71, 247–258 10.1016/j.cardiores.2006.05.001 16765337

[B58] Peach M. J. (1977). Renin-angiotensin system: biochemistry and mechanisms of action. Physiol. Rev. 57, 313–370 191856 10.1152/physrev.1977.57.2.313

[B59] Peterson J. R. Burmeister M. A. Tian X. Zhou Y. Guruju M. R. Stupinski J. A. (2009). Genetic silencing of Nox2 and Nox4 reveals differential roles of these NADPH oxidase homologues in the vasopressor and dipsogenic effects of brain angiotensin, II. Hypertension 54, 1106–1114 10.1161/HYPERTENSIONAHA.109.140087 19805637 PMC2773438

[B60] Prewitt R. L. Dobrian A. D. Schriver S. D. (2001). Role of Angiotensin II and free radicals in blood pressure regulation in a rat model of renal hypertension. Hypertension 38, 361–366 10.1161/01.HYP.38.3.361 11566905

[B61] Queiroz T. M. Guimarães D. D. Mendes-Júnior L. G. Braga V. A. (2012). α-Lipoic acid reduces hypertension and increases baroreflex sensitivity in renovascular hypertensive rats. Molecules 17, 13357–13367 10.3390/molecules171113357 23143148 PMC6268197

[B62] Queiroz T. M. MacHado N. T. Furtado F. F. Oliveira-Filho A. A. Alustau M. C. Figueiredo C. S. (2011). Vasorelaxation induced by *Dictyota pulchella* (Dictyotaceae), a brown alga, is mediated via inhibition of calcium influx in rats. Mar. Drugs 9, 2075–2088 10.3390/md9102075 22073010 PMC3210619

[B63] Rajagopalan S. Kurz S. Munzel T. Tarpey M. Freeman B. A. Griendling K. K. (1996). Angiotensin II-mediated hypertension in the rat increases vascular superoxide production via membrane NADH/NAD(P)H oxidase activation: contribution to alterations of vasomotor tone. J. Clin. Investig. 97, 1916–1923 10.1172/JCI118623 8621776 PMC507261

[B64] Sadjadi J. Puttaparthi K. Welborn M. B. Rogers T. E. Moe O. Clagett G. P. (2002). Upregulation of autocrineparacrine renin-angiotensin systems in chronic renovascular hypertension. J. Vasc. Surg. 36, 386–392 10.1067/mva.2002.125016 12170196

[B65] Santos R. A. S. Silva A. C. S. Maric C. Silva D. M. R. MacHado R. P. de Buhr I. (2003). Angiotensin-(1–7) is an endogenous ligand for the G protein coupled receptor Mas. Proc. Natl. Acad. Sci. U.S.A. 100, 8258–8263 10.1073/pnas.1432869100 12829792 PMC166216

[B66] Stegbauer J. Coffman T. M. (2011). New insights into angiotensin receptor actions: from blood pressure to aging. Curr. Opin. Nephrol. Hypertens. 20, 84–88 10.1097/MNH.0b013e3283414d40 21076298 PMC3087382

[B67] Sun C. Zubcevic J. Polson J. W. Potts J. T. Diez-Freire C. Zhang Q. (2009). Shift to an involvement of phosphatidylinositol 3-kinase in angiotensin II actions on nucleus tractus solitarii neurons of the spontaneously hypertensive rat. Circ. Res. 105, 1248–1255 10.1161/CIRCRESAHA.109.208926 19850939 PMC2810537

[B68] Touyz R. M. Yao G. Schiffrin E. L. (2003). c-Src induces phosphorylation and 533translocation of p47phox: role in superoxide generation by angiotensin II in human 534 vascular smooth muscle cells. Arterioscler. Thromb. Vasc. Biol. 23, 981–987 10.1161/01.ATV.0000069236.27911.68 12663375

[B69] Turko I. V. Murad F. (2002). Protein nitration in cardiovascular diseases. Pharmacol. Rev. 54, 619–634 12429871 10.1124/pr.54.4.619

[B70] Ungvari Z. Csiszar A. Kaminski P. M. Wolin M. S. Koller A. (2004). Chronic high pressure-induced arterial oxidative stress: involvement of protein kinase C-dependent NAD(P)H oxidase and local renin-angiotensin system. Am. J. Pathol. 165, 219–226 10.1016/S0002-9440(10)63290-7 15215177 PMC1618527

[B71] Wolin M. S. (2005). Loss of vascular regulation by soluble guanylate cyclase is emerging as a key target of the hypertensive disease process. Hypertension 45, 1068–1069 10.1161/01.HYP.0000165675.94771.38 15883231

[B72] Wu L. De Champlain J. (1999). Effects of superoxide on signaling pathways in smooth muscle cells from rats. Hypertension 34, 1247–1253 10.1161/01.HYP.34.6.1247 10601126

[B73] Xu P. Sriramula S. Lazartigues E. (2011). ACE2/ANG-(1-7)/Mas pathway in the brain: the axis of good. Am. J. Physiol. Regul. Integr. Comp. Physiol. 300, 804–817 10.1152/ajpregu.00222.2010 21178125 PMC3075080

[B74] Xue B. Beltz T. G. Johnson R. F. Guo F. Hay M. Johnson A. K. (2012). PVN adenovirus-siRNA injections silencing either NOX2 or NOX4 attenuate aldosterone/NaCl-induced hypertension in mice. Am. J. Physiol. Heart Circ. Physiol. 302, H733–H741 10.1152/ajpheart.00873.2011 22140041 PMC3353786

[B75] Zimmerman M. C. Dunlay R. P. Lazartigues E. Zhang Y. Sharama R. V. Engelhardt J. F. (2004a). Requirement for Rac 1-dependent NADPH oxidase in the cardiovascular and dipsogenic actions of angiotensin II in the brain. Circ. Res. 95, 532–539 10.1161/01.RES.0000139957.22530.b9 15271858

[B76] Zimmerman M. C. Lazartigues E. Sharma R. V. Davisson R. L. (2004b). Hypertension caused by angiotensin II infusion involves increased superoxide production in the central nervous system. Circ. Res. 95, 210–216 10.1161/01.RES.0000135483.12297.e4 15192025

[B77] Zimmerman M. C. Lazartigues E. Lang J. A. Sinnayah P. Ahmad I. M. Spitz D. R. (2002). Superoxide mediates the actions of angiotensin II in the central nervous system. Circ. Res. 91, 1038–1045 10.1161/01.RES.0000043501.47934.FA 12456490

